# Metabolomics in Plant Research—From Ecometabolomics to Metabolotyping

**DOI:** 10.3390/cells12040600

**Published:** 2023-02-13

**Authors:** Sara Rinalducci, Stefanie Wienkoop

**Affiliations:** 1 Department of Ecological and Biological Sciences, University of Tuscia, 01100 Viterbo, Italy; 2 Department of Functional and Evolutionary Ecology, University of Vienna, 1030 Vienna, Austria

In this Special Issue, a state-of-the-art review of the current knowledge of sample preparation and LC–MS techniques for the analyses of nucleosides and nucleotides in plants was published [1]. It provides a comprehensive overview of the analytical techniques available for this challenging class of metabolites.

Metabolome profiling is a powerful tool for molecular phenotyping of plant organs and tissues and to discover novel key metabolites and “metabolotypes”. The study of [2] revealed that homocitrate and other metabolites involved in lysine biosynthesis and degradation of papilionoid and mimosoid root nodules infected with the *P. phymatum*, accumulated in all plant nodules and are hence crucial for symbiosis. Through mass spectrometry imaging (MSI) and high-pH anion exchange chromatography with pulsed amperometric detection (HPAEC-PAD), fructoligosaccharides (FOS)/fructopolysaccharides distribution patterns and carbohydrate profiles were resolved by Witzel and Matros in asparagus roots [3], demonstrating an accumulation gradient of fructans from the outer cortex to the inner vascular root tissues. A metabolome comparison of germinating seeds in three annual *Brachypodium* species was performed to explore the impact of allotetraploidization [4]. One review summarizes several areas where metabolomics played a crucial role in defining the phenotype (trait) during crop improvement [5].

Metabolite profiling is also a major tool to comprehensively and unbiasedly investigate various plant stresses and their metabolic reprogramming towards improved tolerance [6,7,8,9]. Here, two studies are dealing with drought stress [6,7] and one with long-term Cd exposure [8]. Skalska and collaborators [6] demonstrated how genomic variation is reflected in the metabolomes. They studied various accessions of *Brachypodium* that were collected from five different climatic regions of Turkey. Distinct metabolotypes were found, which lead to differences in drought tolerance. The role and importance of the spike organs during wheat grain filling and under drought were investigated by Vergara-Diaz and coworkers [7]. The relevance of the analyses of different plant tissues was emphasized by Gutsch and coworkers [8], who found a tissue-specific metabolic stress response, important during acclimation of Cd stress in *Medicago sativa*. Furthermore, one review summarizes recent findings on the effects and roles of metabolites on the plants’ enhanced salt tolerance [9]. Intriguing insights into plant strategies to adapt to changing environments can be offered by ecometabolomic studies. Schweiger et al. [10] explored the composition of primary and specialized metabolites in leaves of twenty woody species from the Mediterranean region when grown under common garden conditions, demonstrating highly species-specific leaf metabolomes in addition to some phylogenetic imprints.

The profiling of phytochemical composition in medicinal plants and health-beneficial vegetables for the human diet is another powerful application of metabolomics. Thus, Siddaiah et al. [11] performed an extensive metabolite profiling of both organic and aqueous extracts from *Alangium salviifolium* bark, a plant traditionally used to treat several diseases; whereas, Bielecka and coworkers [12] combined metabolomics with a DNA barcoding approach to acquire information about phytochemical and genetic diversity of two traditional Asian medicinal and aromatic species of *Salvia* subg. *Perovskia*. Finally, the research group of Dr. Pocsfalvi [13] demonstrated that micro- and nanosized vescicles (MVs and NVs, respectively) from four Citrus species specifically inhibit the proliferation of lung, skin and breast cancer cells, with no substantial effect on the growth of non-malignant cells. GC-MS-based metabolomics revealed distinct metabolite profiles in grapefruit-derived NVs and MVs, supporting their potential in new anticancer strategies. Overall, these studies confirmed the possible use of herbal remedies as an alternative to modern medicine in treating several ailments. Koudela et al. [14], instead, measured metabolomic profiles of four newly bred carrot cultivars used for commercial production in central and eastern Europe to investigate the effect of different farming systems on the levels of health-related compounds.
